# Unveiling myocardial microstructure shifts: exploring the impact of diabetes in stable CAD patients through CMR T1 mapping

**DOI:** 10.1186/s13098-024-01395-9

**Published:** 2024-07-09

**Authors:** Gustavo André Boeing Boros, Whady Hueb, Paulo Cury Rezende, Carlos Eduardo Rochitte, Cesar Higa Nomura, Eduardo Gomes Lima, Matheus de Oliveira Laterza Ribeiro, Anderson Roberto Dallazen, Rosa Maria Rahmi Garcia, Jose Antonio Franchini Ramires, Roberto Kalil-Filho

**Affiliations:** 1 Department of Clinical Cardiology - Heart Institute (InCor), University of São Paulo, São Paulo, Brazil; 2 Heart Institute (InCor), University of São Paulo, São Paulo, Brazil; 3 Divisão Clínica - Instituto do Coração (InCor), Faculdade de Medicina, Hospital das Clínicas - HCFMUSP, Universidade de São Paulo, Av. Dr. Enéas de Carvalho Aguiar 44, AB 1, Sala 114, Cerqueira César, São Paulo, 05403–000 SP Brazil

**Keywords:** Type 2 diabetes mellitus, Coronary artery disease, T1 mapping, Cardiac magnetic resonance - feature tracking, Extracellular volume

## Abstract

**Background:**

This study investigates myocardial structural changes in stable coronary artery disease (CAD) patients with type 2 diabetes (T2D) using cardiac magnetic resonance (CMR) strain and T1 mapping.

**Methods:**

A total of 155 stable CAD patients underwent CMR examination, including left ventricular (LV) morphology and function assessment, late gadolinium enhancement (LGE), and feature tracking (CMR-FT) for LV global longitudinal, circumferential, and radial strain. T1 mapping with extracellular volume (ECV) evaluation was also performed.

**Results:**

Among the enrolled patients, 67 had T2D. Diabetic patients exhibited impaired LV strain and higher ECV compared to non-diabetics. Multivariate analysis identified T2D as an independent predictor of increased ECV and decreased strain.

**Conclusions:**

CMR-based strain and T1 mapping highlighted impaired myocardial contractility, elevated ECV, and potential interstitial fibrosis in diabetic patients with stable CAD. This suggests a significant impact of diabetes on myocardial health beyond CAD, emphasizing the importance of a comprehensive assessment in these individuals.

**Trial registration:**

http://www.controlled-trials.com/ISRCTN09454308

## Graphical abstract

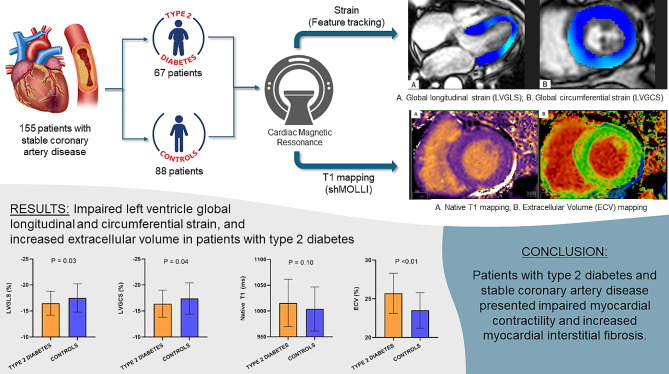

## Introduction

Type 2 diabetes (T2D) is a robust independent cardiovascular risk factor for major cardiac events and significantly increases the likelihood of developing heart failure (HF) [1, 2]. While coronary artery disease (CAD) and ischemic cardiomyopathy remain the primary causes of HF in T2D, other pathophysiological processes contribute to myocardial structural derangements. Hyperglycemia, insulin resistance, neurohormonal dysregulation, autonomic neuropathy, and cellular metabolic disorders collectively form a complex mechanism underlying diabetic cardiomyopathy [3–5]. Early recognition of this disorder allows the use of therapeutic strategies for better clinical management and possible intervention in the progress of the disease.

Cardiac magnetic resonance (CMR), with recent advancements, allows detailed characterization of cardiac structures through both anatomical and functional analysis. CMR tissue tracking technology, compared to echocardiography, offers access to deformation parameters in post-processed cine images with simpler application and improved image quality [6, 7]. Furthermore, T1 relaxation time mapping provides a non-invasive approach to assess both cellular and interstitial compartments, enabling unique pathological correlation [8]. Extracellular volume (ECV) evaluation can detect early myocardial tissue changes, reflecting potential interstitial matrix alterations or diffuse fibrosis [9–11]. Studies evaluating tissue changes in T2D have shown impaired left ventricular global longitudinal strain in non-ischemic diabetic patients compared to controls [12, 13]. However, findings regarding native T1 and ECV values in diabetic patients with preserved left ventricular ejection fraction (LVEF) compared to normal controls remain inconsistent, particularly due to the lack of comprehensive CAD assessment [14–18].

We hypothesize that T2D, independent of CAD, is associated with structural myocardial changes, and that CMR strain and T1 mapping can identify these alterations. Therefore, we aim to study patients with stable coronary artery disease with and without type 2 diabetes.

## Research design and methods

This subanalysis delves into the comprehensive dataset generated by the prospective trial titled “Accuracy of Myocardial Biomarkers in the Diagnosis of Myocardial Infarction After Revascularization as Assessed by Cardiac Resonance: The Medicine, Angioplasty, Surgery Study V (MASS-V)”. Details on study design and protocol are published elsewhere [19]. In summary, a total of 202 patients were enrolled, meeting the criteria of having multivessel CAD with preserverd LVEF, and an indication for coronary artery bypass grafting or percutaneous coronary intervention. Individuals with recent (< 6 months) myocardial infarction, overt or suspected infections, active rheumatologic diseases, chronic renal failure (creatinine > 2.0 mg/dL), recent (< 6 months) pulmonary embolism or venous thromboembolism, and contraindications to CMR, such as pacemaker implantation or severe claustrophobia, were excluded from the study. All enrolled patients underwent CMR within six days before the revascularization procedure. The study was conducted in accordance with the principles outlined in the Helsinki Declaration and received approval from the institutional ethics committee at the Heart Institute of the University of São Paulo Medical School, São Paulo, Brazil. Informed consent was obtained from all participants. Out of the original 202 patients, a subset of 155 individuals qualified for this subanalysis, with 35 excluded due to incomplete T1 mapping phases acquisition and 12 experiencing CMR image artifacts hindering proper analysis. The sample size was linked to the MASS-V trial and thus not specifically calculated for this subanalysis. However, we estimated a medium effect size (Cohen’s d = 0.5) based on previous studies on diabetic cardiomyopathy and related treatments.

**Fig. 1:**
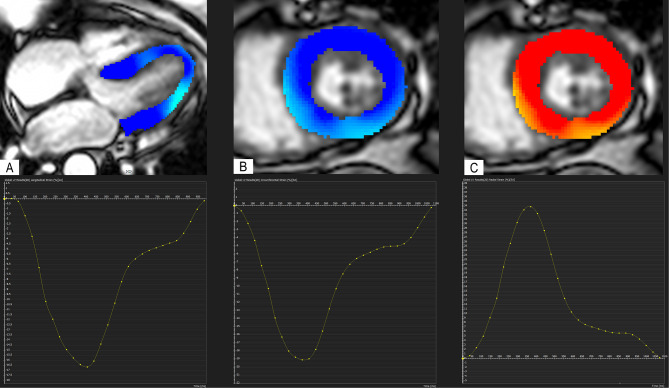
Representative strain images and curves of a diabetic patient. Left ventricular global longitudinal strain **(A)**, left ventricular global circumferential strain **(B)**, and left ventricular global radial strain **(C)**

**Fig. 2:**
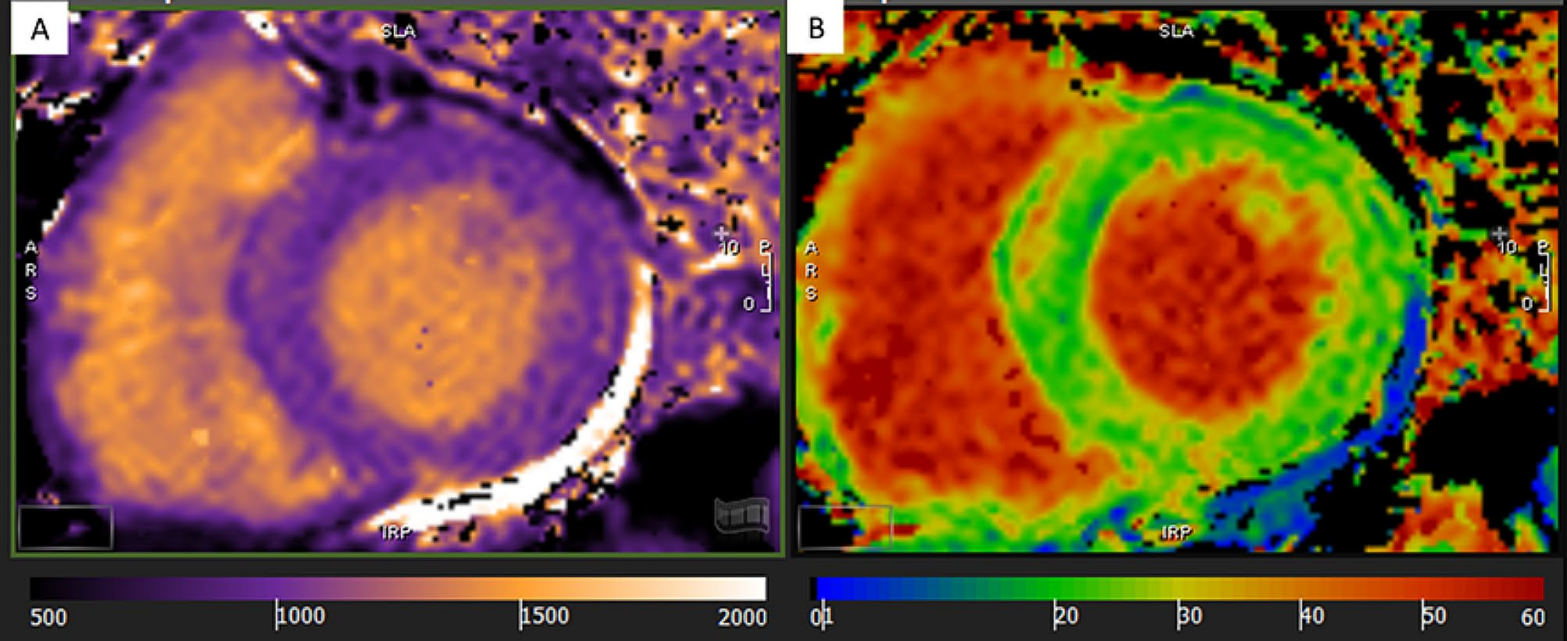
Representative color maps of a non-diabetic patient at the left ventricle middle short-axis slice. Native T1 mapping **(A)** and extracellular volume (ECV) mapping **(B)**

CMR examinations were conducted using a 1.5 Tesla Philips Achieva^®^ scanner equipped with a dedicated 5-element phased-array cardiac surface coil, ensuring high-quality imaging. Electrocardiogram (ECG) synchronization was employed throughout the imaging process. Acquisition protocols followed current guidelines from the Society for Cardiovascular Magnetic Resonance [20, 21]. Standard steady-state free precession (SSFP) cine sequences were acquired in both short (slice thickness: 8 mm) and long axes (2-, 3- and 4-chamber) of the left ventricle, capturing 30 cardiac phases to achieve sub-50 ms temporal resolution. Parameters such as left ventricular end-diastolic volume (LVEDV), left ventricular end-systolic volume (LVESV), LVEF, and left ventricular mass (LVM) were measured and indexed to body surface area (BSA). Late gadolinium enhancement (LGE) imaging was performed using phase-sensitive inversion recovery (PSIR). CMR feature tracking (CMR-FT) was conducted using short-axis cine images and 2- and 4-chamber long-axis images. Manual delineation of end-diastolic left ventricular endocardial and epicardial contours in all images was followed by automated tracking, enabling the calculation of left ventricular global longitudinal strain (LVGLS), left ventricular global circumferential strain (LVGCS), and left ventricular global radial strain (LVGRS) (Fig. 1). T1 mapping images were acquired utilizing the Shortened modified Look-Locker Inversion recovery (ShMOLLI) technique in three short-axis slices (basal, middle, and apical). These images provided native T1, post-contrast T1, and extracellular volume (ECV) values (Fig. 2) [22, 23]. Native T1 was obtained before the administration of gadolinium-based contrast, while post-contrast T1 was acquired 15–20 min after intravenous injection of gadoterate meglumine (0.1 mmol/kg of body weight). ECV was calculated using the formula: ECV = λ x (1-hematocrit). The partition coefficient (λ) was determined as λ = ΔR1myocardium/ΔR1blood, where ΔR1 represents the difference in relaxation rates (1/T1) before and after contrast administration. Notably, only segments without LGE were included in native T1, post-contrast T1, and ECV assessment. Trabeculae and papillary muscles were excluded from myocardial evaluation. All analyses were performed offline using dedicated commercial software (Cvi42, Circle Cardiovascular Imaging, Calgary, AB, Canada) by two blinded observers. Discrepancies were resolved through consensus, or consultation with a third blinded observer if necessary.

### Statistical analysis

Continuous variables were described using means and standard deviations (SD) for normally distributed data or medians and interquartile ranges (IQR) for non-normal data. Categorical variables were presented as frequencies and percentages.

Comparisons between groups for categorical variables were performed using chi-squared tests, Fisher’s exact tests, or likelihood ratio tests, depending on the number of expected cells per category. The Shapiro-Wilk test assessed normality of distribution for continuous variables. Normally distributed data were compared using Student’s t-tests, while Mann-Whitney U tests were used for non-normal data. Univariate associations were evaluated with Pearson or Spearman correlation coefficients, depending on data normality.

Multivariate linear regression analysis explored the relationship between native T1, ECV, LVGLS, LVGCS, LVGRS, and the presence of T2D, additionally considering other relevant factors. Variables included in the model were clinically relevant or demonstrated statistical significance (*P* < 0.2 in univariate analysis).

All analyses were conducted using R software (version 3.6.2), with statistical significance set at *P* < 0.05.

## Results

**Fig. 3:**
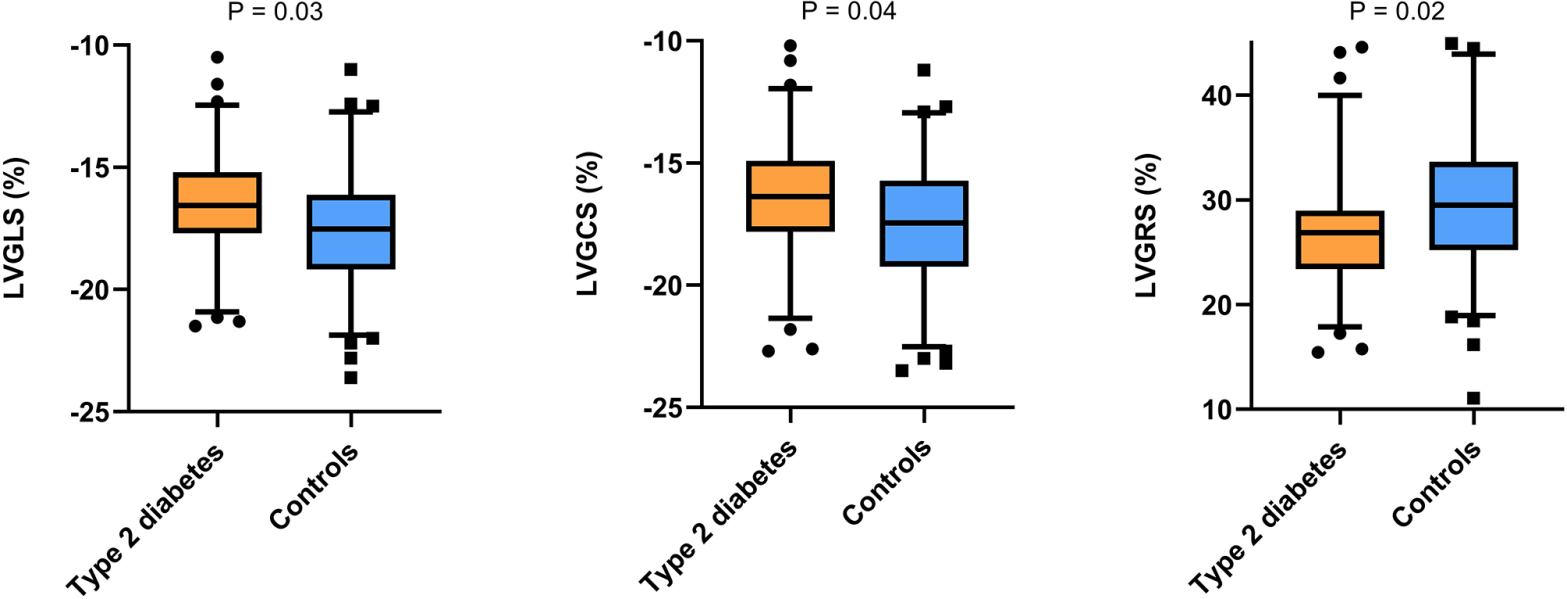
Box plots comparing left ventricular global longitudinal (LVGLS), circumferential (LVGCS) and radial (LVGRS) strain in type 2 diabetes and controls patients

**Fig. 4:**
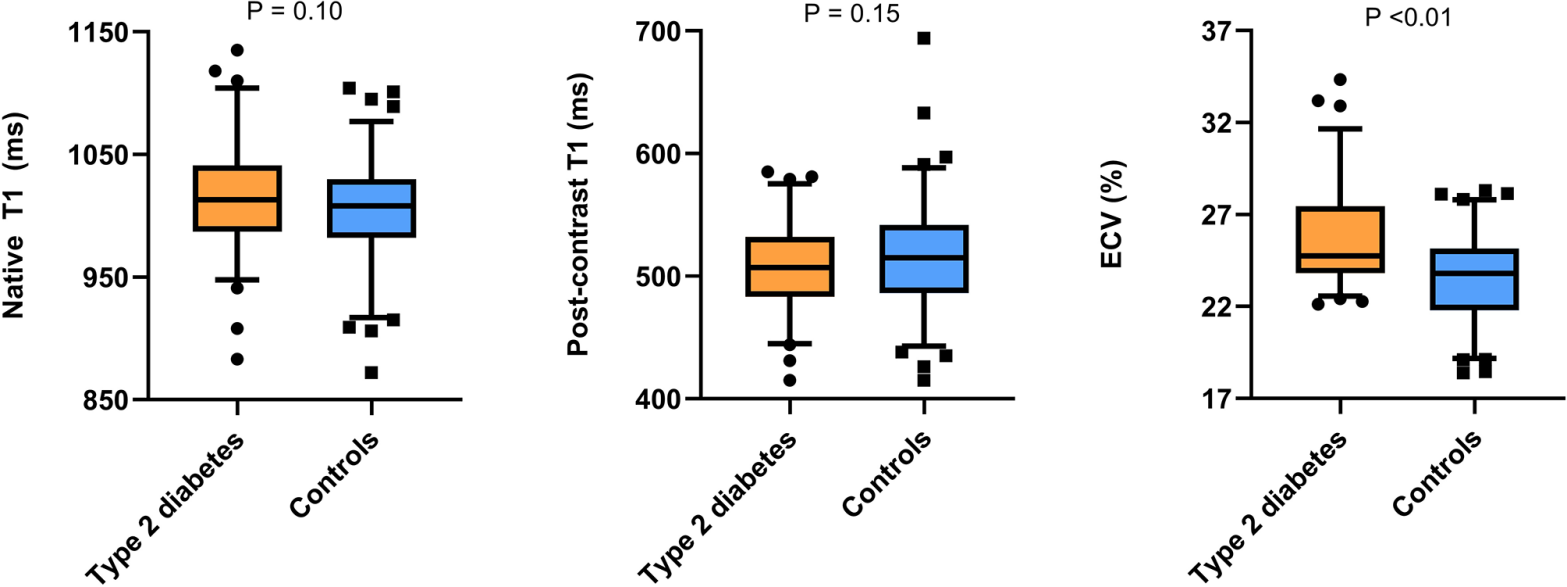
Box plots comparing native T1, post-contrast T1, and extracellular volume (ECV) in type 2 diabetes and controls patients

Baseline characteristics of the 155 patients are summarized in Table 1. Patients were classified as either T2D (*n* = 67) or controls (*n* = 88). While clinical, laboratory, and angiographic data were generally similar between groups, T2D patients had a lower prevalence of current or past smoking (20% vs. 33%, *P* = 0.03) and significantly higher hemoglobin A1c levels (7.7 ± 1.2% vs. 5.3 ± 0.7%, *P* < 0.01). CMR analysis on left ventricular volumes, mass, and LVEF did not differ between T2D and control groups (Table 2). However, T2D patients exhibited significantly lower global longitudinal strain (LVGLS), circumferential strain (LVGCS), and radial strain (LVGRS) compared to controls (-16.5% ± 2.3% vs. -17.5% ± 2.7%, *P* = 0.03; -16.4% ± 2.6% vs. -17.4% ± 3.0%, *P* = 0.04; 26.9% ± 5.9% vs. 29.5% ± 7.2%, *P* = 0.02, respectively) (see Fig. 3 for illustration). Despite similar native T1 and post-contrast T1 values between groups, T2D patients had significantly higher ECV (25.7% ± 2.6% vs. 23.5% ± 2.3%, *P* < 0.01) (see Fig. 4 for illustration). Relationship between CMR parameters and T2D in univariate analysis revealed no significant associations between native T1 or ECV and LVGLS, LVGCS, or LVGRS. Multivariate linear regression analysis, adjusting for clinically relevant covariates (age, sex, BMI, hypertension) and statistically significant covariates (heart rate, smoking status, creatinine, LVEF), confirmed that T2D was an independent predictor for increased ECV (β = 2.24, *P* < 0.01), impaired LVGLS (β = 0.72, *P* = 0.02), impaired LVGCS (β = 0.82, *P* = 0.01), and impaired LVGRS (β = -2.22, *P* < 0.01) (Table 3). As expected, LVEF was also correlated with reduced LVGLS (β = -0.19, *P* < 0.01), LVGCS (β = -0.23, *P* < 0.01), and LVGRS (β = 0.52, *P* < 0.01).

**Table 1:** Baseline characteristics of the study population

	T2D patients (*n* = 67)	Controls (*n* = 88)	*P* value
Age, yrs	62 ± 9	62 ± 10	0.57
Male, (%)	48 (72)	58 (66)	0.45
BMI, kg/m2	28.8 ± 4.2	27.4 ± 4.1	0.05
BSA, m2	1.8 ± 0.4	1.8 ± 0.2	0.57
Heart rate, bpm	60 ± 8	57 ± 7	0.01
Current or past smoker, (%)	12 (20)	29 (33)	0.03
Hypertension, (%)	59 (88)	73 (83)	0.38
Previous myocardial infarct, (%)	23 (34)	30 (34)	0.97
3-vessel CAD, (%)	48 (72)	55 (63)	0.23
SYNTAX score	20 ± 8	20 ± 9	0.77
LABORATORY			
Hematocrit, %	43 ± 4	43 ± 4	0.80
Creatinine, mg/dl	1.07 ± 0.2	1.02 ± 0.2	0.17
Cholesterol, mg/dl	171 ± 47	175 ± 48	0.55
LDL, mg/dl	99 ± 36	105 ± 39	0.42
HDL, mg/dl	38 ± 11	38 ± 13	0.86
Triglyceride, mg/dl	167 ± 110	167 ± 103	0.99
Hemoglobin A1C, %	7.7 ± 1.2	5.3 ± 0.7	< 0.01
MEDICATIONS			
Acetylsalicylic acid, (%)	67 (100)	88 (100)	1
Statin, (%)	66 (98)	84 (95)	0.77
Other hypolipidemic drug, (%)	7 (10)	9 (10)	0.97
Beta blocker, (%)	60 (89)	80 (90)	0.86
ACE inhibitor, (%)	37 (55)	44 (50)	0.22
Angiotensin receptor blocker, (%)	23 (34)	28 (32)	0.42
Calcium channel blocker, (%)	20 (30)	30 (24)	0.38
Metformin, (%)	54 (81)	4 (4)	< 0.01
Sulfonylurea, (%)	29 (43)	-	
Insulin, (%)	22 (33)	-	

Abbreviations: BMI, body mass index; BSA, body surface area; CAD, coronary artery disease; LDL, low density lipoprotein; HDL, high density lipoprotein; ACE, angiotensin-converting enzyme

**Table 2:** Cardiac magnetic resonance findings

	T2D (*n* = 67)	Controls (*n* = 88)	*P* value
LVEDVi, ml/m2	74.3 ± 16.0	73.0 ± 16.3	0.45
LVESVi, ml/m2	39.9 ± 8.3	40.9 ± 9.9	0.54
LVEF, %	54.9 ± 9.6	56.2 ± 9.0	0.42
LVMi, g/m2	53.4 ± 11.4	51.7 ± 11.8	0.40
LVGLS, %	-16.5 ± 2.3	-17.5 ± 2.7	0.03
LVGCS, %	-16.4 ± 2.6	-17.4 ± 3.0	0.04
LVGRS, %	26.9 ± 5.9	29.5 ± 7.2	0.02
Native T1, ms	1015.5 ± 46.0	1003.8 ± 42.8	0.10
Post-contrast T1, ms	507.3 ± 35.9	516.8 ± 44.5	0.15
ECV, %	25.7 ± 2.6	23.5 ± 2.3	< 0.01

Abbreviations: LVEDVi, left ventricular end-diastolic volume indexed to body surface area; LVESVi, left ventricular end systolic volume indexed to body surface area; LVEF, left ventricular ejection fraction; LVMi, left ventricular mass indexed to body surface area; LVGLS, left ventricular global longitudinal strain; LVGCS, left ventricular global circumferential strain; LVGRS, left ventricular global radial strain; ECV, extracellular volume

**Table 3:** Multivariate adjusted analysis of T1 mapping and strain

	Native T1			Post-contrast T1			ECV			LVGLS			LVGCS			LVGRS	
	β	*P* value		β	*P* value		β	*P* value		β	*P* value		β	*P* value		β	*P* value
T2D	11.47	0.13		-11.57	0.09		2.24	< 0.01		0.72	0.02		0.82	0.01		-2.22	< 0.01
Age	0.27	0.52		-0.03	0.94		0.02	0.36		0.00	0.88		-0.01	0.39		0.07	0.13
Sex	-8.59	0.33		20.52	< 0.01		-1.17	0.02		0.50	0.16		0.64	0.07		-1.68	0.06
BMI	-1.05	0.25		-2.06	0.01		-0.08	0.12		0.07	0.06		-0.01	0.70		0.04	0.65
Hypertension	-5.24	0.62		1.68	0.86		0.34	0.57		0.08	0.86		-0.28	0.51		1.13	0.29
Heart rate	0.70	0.16		1.10	0.01		0.02	0.56		-0.04	0.06		0.00	0.83		0.01	0.81
Current or past smoker	4.46	0.60		0.22	0.98		0.07	0.88		0.24	0.49		0.51	0.13		-1.25	0.15
Creatinine	12.95	0.44		-40.59	< 0.01		0.31	0.74		0.46	0.50		-0.04	0.95		-0.48	0.78
LVEF	0.21	0.60		-0.37	0.31		-0.02	0.51		-0.19	< 0.01		-0.23	< 0.01		0.52	< 0.01

Abbreviations: ECV, extracellular volume; LVGLS, left ventricular global longitudinal strain; LVGCS, left ventricular global circumferential strain; LVGRS, left ventricular global radial strain; T2D, type 2 diabetes; BMI, body mass index; LVEF, left ventricular ejection fraction

## Discussion

The present study demonstrated that patients with type 2 diabetes exhibit decreased strain and higher extracellular volume values in a stable coronary artery disease setting, compared to controls, without notable differences in left ventricular structural parameters assessed by cardiac magnetic resonance. This association persisted even after adjusting for potential covariates. These findings may have implications for understanding and detecting diabetic cardiomyopathy.

In T2D, myocardial tissue is susceptible to higher rates of myocyte necrosis, collagen deposition, and interstitial fibrosis. Multiple factors contribute to the development of progressive myocardial damage and subsequent fibrosis, including hyperglycemia, increased oxidative stress, fatty acid availability, and activation of the renin-angiotensin-aldosterone system [3, 28]. By utilizing CMR feature tracking and T1 mapping, we explored potential and accessible techniques for identifying patients at risk for adverse LV remodeling.

It is widely demonstrated that diabetes causes interstitial fibrosis and microvascular dysfunction. A general understanding is that these are the underlying factors causing diastolic dysfunction, which is highly prevalent in patients with T2D [29].

Our findings revealed impaired LV global longitudinal strain (LVGLS), LV global circumferential strain (LVGCS), and LV global radial strain (LVGRS) in diabetic patients, with no significant differences in mass or volumes, independently of T1 mapping analysis. These abnormalities in myocardial contractility may indicate a higher risk of heart failure development, as LV strain can be employed to detect early subclinical myocardial dysfunction. We prioritized global strain evaluation instead of regional assessment due to their greater validation and reproducibility in our population [30, 31]. It is known that there is significant overlap in strain values between LGE and non-LGE areas, particularly in cases with LVEF > 40%, as well as influence of multiple factors must be considered, such as LV remodeling and the time elapsed since the previous myocardial infarction [31, 32].

Although not statistically significant, there was a trend toward higher native T1 and lower post-contrast T1 in the myocardium of diabetic patients. As ECV depends on pre- and post-contrast T1 in the tissue, hematocrit, and pre- and post-contrast T1 in the blood, it can amalgamate slight differences in T1 values and better reflect changes in the extracellular matrix. Notably, higher ECV is associated with increased collagen volume fraction and myocardial fibrosis in histological comparison [33, 34]. LGE areas were excluded in the ECV analysis in this CAD patients study to assess only interstitial fibrosis in otherwise normal tissue. Furthermore, stable CAD is not known to affect the interstitial matrix in the absence of infarct.

While recent study demonstrated correlation between higher ECV and lower strain values in diabetic patients without clinical evidence of CAD, in our sample we did not find this direct association [18]. This suggests the contribution of other factors that impair myocardial contractility besides the increased ECV, given that subtle differences in LVEF and LGE are known to further reduce LV strain [24, 26].

Overall, our results emphasize that T2D patients have impaired myocardial contractility and increased myocardial interstitial fibrosis compared to matched controls. This could be used as a potential early assessment of adverse left ventricle remodeling in these patients, as well as guide therapy strategies [25].

However, this study has limitations. Firstly, it was a single-center study with 155 subjects, a substantial number for CMR studies but possibly considered small for certain statistical analyses. Secondly, CMR has undergone extensive study in recent years, but differences in magnetic strengths, acquisition protocols, contrast agent, and standardized methods can yield varied results. We attempted to mitigate these limitations by selecting a homogeneous study sample. Thirdly, the addition of T2 mapping could enhance the assessment of myocardial inflammation and edema, data not available in our sample because of different acquisition protocols. Furthermore, while the inclusion criteria specifically targeted patients with preserved LVEF, it’s crucial to acknowledge the limitation posed by the absence of specific data on diastolic dysfunction from echocardiography. Finally, being a cross-sectional study, inherent limitations are present, and a prospective study with clinical follow-up could provide additional data to support the development of diabetic cardiomyopathy following our results.

## Conclusion

In this study, diabetic patients, when compared to control subjects with stable coronary artery disease, presented impaired myocardial contractility, with decreased left ventricular global strain (longitudinal, circumferential, and radial), as well as increased myocardial interstitial fibrosis assessed by T1 mapping ECV.

## Data Availability

No datasets were generated or analysed during the current study.

## Abbreviations

CAD Coronary artery disease {next line} CMR: cardiac magnetic resonance
CMR-FT: cardiac magnetic resonance feature tracking
ECV: extracellular volume
LGE: late gadolinium enhancement
LVEDV: left ventricular end-diastolic volume
LVEF: left ventricular ejection fraction
LVESV: left ventricular end-systolic volume
LVGCS: left ventricular global circumferential strain
LVGLS: left ventricular global longitudinal strain
LVGRS: left ventricular global radial strain
T2D: Type 2 diabetes
